# Titanium-Alloy Anchoring System as a Suitable Method of Extracapsular Repair

**DOI:** 10.3389/fvets.2020.592742

**Published:** 2020-12-17

**Authors:** Christopher Dominic, Otto I. Lanz, Noelle Muro, Dominique Sawyere, Karanvir Aulakh, Theresa Pancotto, David Seda

**Affiliations:** ^1^Department of Small Animal Clinical Sciences, Virginia-Maryland College of Veterinary Medicine, Virginia Polytechnic Institute and State University, Blacksburg, VA, United States; ^2^Veterinary Clinical Sciences, Louisiana State University School of Veterinary Medicine, Louisiana State University, Baton Rouge, LA, United States; ^3^Aella SA, Zurich, Switzerland

**Keywords:** cranial cruciate ligament, canine, ruby, stifle, extracapsular

## Abstract

**Objective:** To characterize the effect of a titanium-alloy anchoring system (TAS) on the motion of the cranial cruciate ligament (CrCL) deficient stifle. To compare the motion with the TAS to that of the CrCL-intact and CrCL-deficient stifle.

**Study Design:** Each canine pelvic limb was mounted in a loading jig under 30% body weight. Motion data was collected using an electromagnetic tracking system at stifle angles of 125°, 135°, and 145° with the CrCL-intact, CrCL-deficient and the TAS applied.

**Results:** Total translation of the CrCL-deficient stifle following the TAS was reduced, but remained greater than the CrCL-intact stifle at angles of 125°, 135°, and 145°. Internal rotation of the TAS groups was greater than the CrCL-intact group at 145°, but not 125° and 135°. Varus motion of the TAS group was decreased compared to the CrCL-deficient group, but increased compared to the CrCL-intact group at angles of 125°, 135°, and 145°.

**Conclusion:** Total translation and internal rotation of the CrCL-deficient stifle following the TAS differed from that of the CrCL-intact stifle. However, the TAS reduced total translation and internal rotation of the tibia relative to the femur in the CrCL-deficient stifle to levels that may yield clinically acceptable results.

## Introduction

The cranial cruciate ligament (CrCL) is an essential structure in maintaining stifle function. The functions of the CrCL include preventing cranial translation and internal rotation of the tibia relative to the femur, counteracting stifle hyperextension, and inhibiting excessive joint motion by allowing coordinated contraction and relaxation of the thigh musculature via proprioceptive fibers (1, 2). Injury to the CrCL leads to lameness, altered joint kinematics, and the progression of osteoarthritis (OA) within the affected joint (3–8).

A common method of extracapsular stifle stabilization is the lateral suture technique. Proposed benefits of the lateral suture compared to osteotomy procedures for CrCL disease include decreased invasiveness, gravity of post-operative complications, and financial cost to owners (9). Numerous complications of the lateral suture techniques are reported and include bone anchor pull out, suture elongation, suture abrasion leading to implant instability or failure (10–14). The overall complication rate for lateral suture technique ranges from 15.5 to 17.4%, with 7.2% of cases requiring revision surgery (11, 15).

The titanium-alloy anchor system^1^ (TAS) examined in this study is an extracapsular method of repair, which shares many of the same benefits as a lateral suture compared to an osteotomy procedure. The TAS aims to restore normal stifle stability using modified titanium alloy bone anchors with pre-attached ultra-high molecular weight polyethylene (UHMWPE) fiber loops of a specified length, and a titanium alloy link. Various lengths of the UHMWPE loops and titanium links are available, and the specific combination of bone anchors and titanium links used is dependent on intraoperative measurements relating to the distance between anchor sites. Proposed advantages over other extracapsular techniques have previously been reported and include superior osseous integration associated with titanium implants and increased strength and resistance to abrasion of UHMWPE (16–19). A clinical study examining 17 patients undergoing the TAS procedure found promising results with improved lameness scores and a low complication rate; however, studies examining the effects of the TAS on tibial translation and rotation in a CrCL-deficient stifle are lacking (19).

The aim of the present study was to compare the translation and internal rotation of the CrCL-deficient stifle stabilized with the TAS to that of the CrCL-intact and CrCL-deficient stifles. We hypothesized that the TAS would reduce translation and internal rotation of the tibia relative to the femur, and the translation and internal rotation of the CrCL-intact and TAS stifles would not significantly differ.

## Materials and Methods

### Specimen Collection and Preparation

Twelve pelvic limbs were harvested from seven skeletally mature cadavers via disarticulation of the hip. Body weights were recorded prior to limb collection. All canines were euthanized for reasons unrelated to this study. The limbs were stored between−23°C and−20°C, and thawed at room temperature for 12–18 h prior to testing. The skin and soft tissues from the head of the femur to the mid-metatarsals were removed, preserving the patella and patellar ligament, the medial and lateral collateral ligaments of the stifle, the medial and lateral menisci, and the cranial and caudal cruciate ligaments. The joint capsule was partially disrupted by removal of the fabellae.

### Radiographic Analysis

Each limb was radiographed to ensure skeletal maturity and rule out the presence of pre-existing orthopedic disease.

### TAS Procedure

The TAS procedure was performed in a similar manner as previously described (19). The bone anchors for each test specimen were positioned at the specific anatomic locations as described below prior to motion testing of each limb. A 1.6 mm Kirschner wire (K-wire) was placed at the cranial eminence of the long digital extensor groove and advanced to exit the proximomedial aspect of the cranial tibial metaphysis. A 3.8 mm diameter cannulated drill bit was used over the previously placed K-wire to create a hole on the lateral tibial cortex. A 3.8 mm counter bore was then used to enlarge the hole to allow placement of a bone anchor. A 1.6 mm K-wire was placed in the femur, level with the distal aspect of the lateral fabella and immediately caudal to the lateral collateral ligament, and was advanced toward the proximal medial femoral trochlear ridge. The appropriate UHMWPE loop length for the femoral anchor was determined by measuring the distance between the end of the tibial anchor UHMWPE loop and the femoral K-wire using a measuring device. The femoral anchor site was then created in a manner identical to the tibial anchor site. The femoral anchor with an appropriate UHMWPE loop length and titanium link size were selected equal the previously measured distance between the end of the tibial anchor UHMWPE loop and femoral anchor site. The titanium link was placed prior to testing of the TAS constructs, after CrCL transection. The tibia was externally rotated to reduce the distance between the UHMWPE loops, and to allow successful placement of the appropriately sized titanium link. The titanium link selected achieved absence of cranial drawer without subsequent external rotation of the tibia relative to the femur.

### Testing Protocol

To simulate the quadriceps muscle unit, a 3.0 mm hole was drilled in the center of the patella in a cranial to caudal direction. UHMWPE fiber was passed through the hole, and the free ends were tied to form a loop, which was attached to one end of a turnbuckle. The opposite end of the turnbuckle was hooked on a 4.0 mm titanium screw placed in the proximal metaphysis of the femur. To simulate the gastrocnemius, a 7-hole 10 ALPS plate^2^ was secured to the caudal aspect of the calcaneus with two 2.7 mm titanium screws. The most proximal hole of the plate was positioned proximal to the calcaneus to allow for attachment of a turnbuckle. A 2.0 mm metal cable was passed through the proximal eyelet of the turnbuckle, and each end of the cable was placed over a 4.0 mm titanium screw located in each of the medial and lateral fabello-femoral articular surfaces of the femur. Two 2.7 mm titanium screws were placed medially in the femur and tibia at the junctions of the proximal and middle, and middle and distal thirds of each bone, to aid the use of a universal goniometer during testing to ensure consistent stifle angle (20).

**Figure 1:**
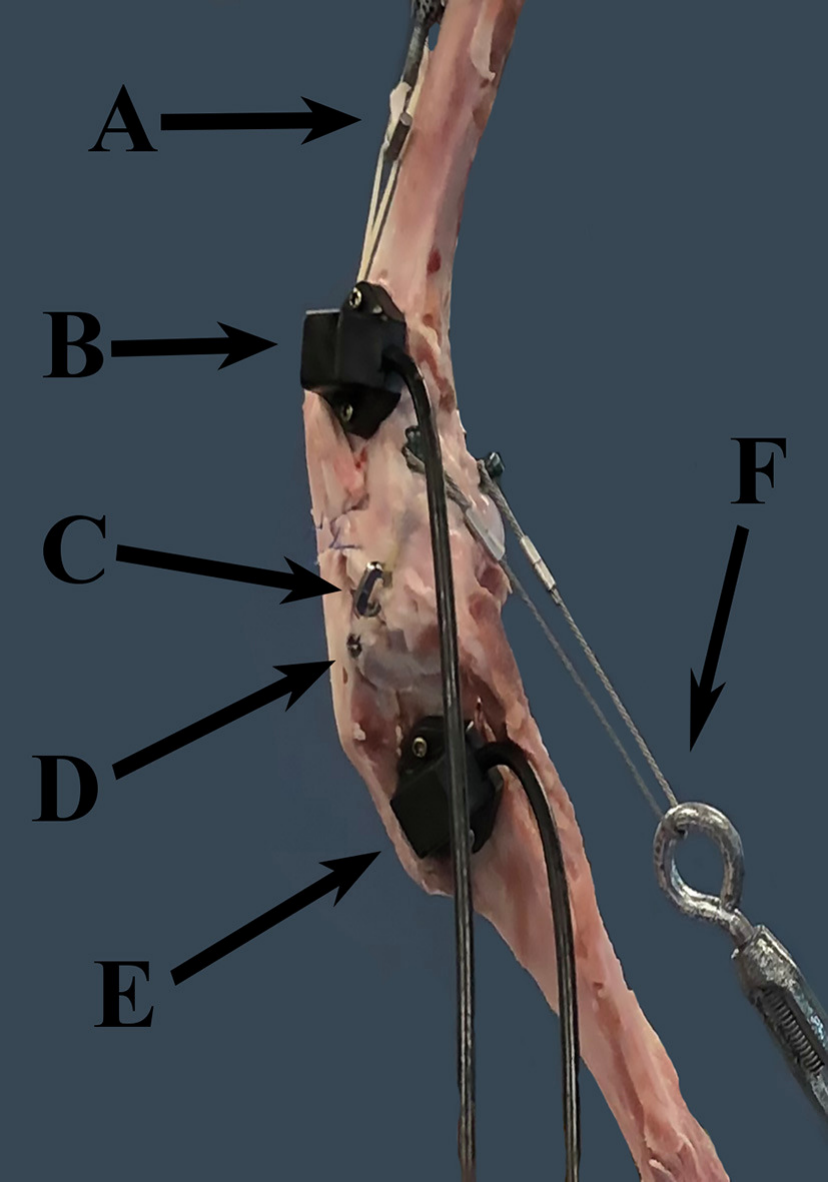
FASTRAK receivers and TAS implants. This figure demonstrates the location of the FASTRAK receivers and TAS implants, which were applied to the lateral aspect of the stifle in each specimen. A = turnbuckle and UHMWPE simulating the quadriceps unit, B = the femoral FASTRAK receiver, C = the titanium link of the TAS, D = the tibial anchor of the TAS, E = the tibial FASTRAK receiver, F = the turnbuckle and metal cable simulating the gastrocnemius. Note: the femoral TAS bone anchor is not visible through the soft tissues in this photo.

Each limb was placed in a custom loading frame for testing. A platform on the frame base was in contact with the paw pads and was covered with 80 grit sandpaper to provide traction during testing. An L-shaped bracket was placed on the platform, attached to a wooden block, that could be aligned with the plantar and medial or lateral aspects of the paw to ensure consistent paw placement. The concave surface of the load cell^3^ was located on the underside of the top portion of the loading frame. This concave surface sat on the head of the femur and allowed contact between the loading frame and limb. Relative motion between the tibia and femur was measured at stifle angles of 145°, 135° and 125°, corresponding to the early, middle, and late stance phase of the gait cycle (21). A universal goniometer was set with each arm along the anatomic long axis of the femur and tibia, along which the titanium screws were placed as a guide. Tracking receivers were placed on the lateral aspect of the limb, one in the distal femoral metaphysis, and one in the proximal tibial metaphysis (Figure 1). Each receiver was connected to the limb via two 2.0 mm titanium screws. The transmitter^4^ for the tracking system was attached to the bar of the loading frame corresponding to the cranial aspect of and level with the stifle.

The stifle angle was set to 145° with appropriate hip and tibio-tarsal angles, 148° and 140°, respectively (21). A static load of 30% of the body weight was applied to the limb to simulate normal hind limb weight bearing, and joint angles were measured a second time for accuracy. Under load, the tibia was translated caudally to collect a baseline data point. The tibia was then released and the limb was allowed to translate and rotate under load, for which a second data point was collected. This process was repeated until six sets of motion data were collected, and the load was removed from the limb. The stifle, hip, and hock angles were adjusted. The protocol was repeated at stifle angles of 135° and 125°. The corresponding hip and tarsal angles were 160° and 145°, and 178° and 155°, respectively. A craniomedial mini-arthrotomy was performed on each stifle to facilitate transection of the CrCL. Complete transection was confirmed by the presence of cranial drawer, and direct visualization. The arthrotomy was closed with 2-0 polydioxanone^5^ in a cruciate pattern. Motion data was collected at each of the three stifle angles with the CrCL transected. The TAS was engaged by placement of the titanium link, and motion data was collected again at each angle.

### Data Collection

**Figure 2:**
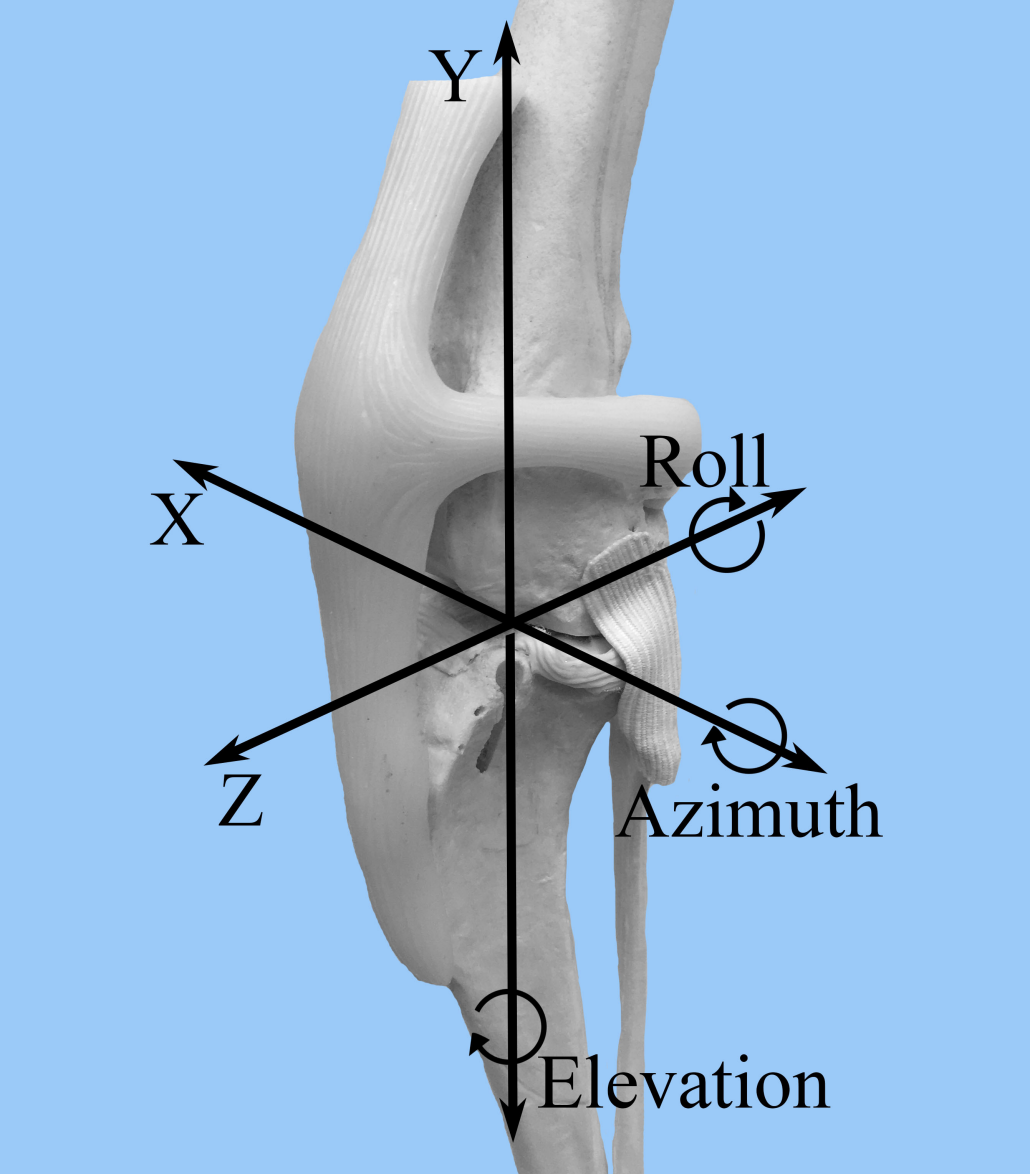
FASTRAK coordinate system. A craniolateral view of the canine stifle is shown with the superimposed coordinate system that corresponds to the direction of stifle motion detected by the FASTRAK Tracking System. Z = cranial-caudal; Y= proximal-distal; X = medial-lateral; Azimuth = flexion-extension; Elevation = internal-external rotation; Roll = varus-valgus.

A FASTRAK^6^ electromagnetic tracking system was used to collect motion data with six degrees of freedom for each limb in each of the described stifle conditions and stifle angles. Data were collected with regard to relative tibial and femoral translation and rotation in the following categories: cranial-caudal (Z), proximal-distal (Y), medial-lateral (X), flexion-extension (azimuth), internal-external rotation (elevation), varus-valgus (roll) (Figure 2). The transmitter was oriented with the x-axis and z-axis horizontal and the y-axis vertical.

In order to calculate relative translational and rotational data of the tibia and femur, baseline data were defined for each individual limb at each of the stifle angles and stifle conditions of interest. This was performed under the applied 30% load to be used in testing conditions. Nine sets of baseline points were collected for each limb. With the limb loaded, the tibia was translated caudally until the caudal cruciate (CaCL) was under tension, and baseline measurements were obtained. Once baseline was established and recorded, the manual tension was released, and the femur and tibia were allowed to translate and rotate under 30% body weight. Relative translation and rotational (azimuth, roll, and elevation) data were calculated in reference to the specific baseline points for that particular limb at the corresponding stifle angle and stifle condition. Motion data were calculated relative to the specific baseline data for each corresponding stifle angle and stifle condition. This was done because the FASTRAK system records the motion of each receiver relative to the transmitter and not to each other. Therefore, establishing baseline data allowed for the relative motion of each receiver to be calculated under the specific testing conditions in reference to a consistent starting point (baseline). This also served as a control to ensure the limb had not significantly shifted within the loading frame between manipulations, which could introduce additional error since the femur or foot were not rigidly fixed.

### Statistical Analysis

Standard error between baseline measurements was calculated for each individual limb at each of the stifle conditions and angles of interest. This was performed to ensure a consistent baseline was established in each loading condition. Total translation was calculated by taking the vector magnitude in the X, Y, and Z planes combined. All tibial rotation data were transformed using a rotation matrix to act as the zero baseline for comparison to femoral rotation. All calculations were performed in MATLAB^7^. Paired *t*-tests and standard error were used to compare the mean values for total translation, internal-external rotation, varus-valgus, and flexion-extension motion. Statistical significance was set as *p*-value < 0.05.

## Results

Three limbs were used to verify testing protocol; nine limbs from five cadavers were included in the final analysis. Mean donor body weight was 25.5 +/- 3.68 kg. All limbs were skeletally mature and free of orthopedic disease based on radiographs. The measured range around each targeted stifle angle for all limbs included in the study was 125° +/- 3°, 135° +/- 3°, and 145° +/- 3°. The measured hip and tarsal angles and associated ranges at stifle angles of 125°, 135° and 145° were 146 ^o^ +/- 5°, 155 ^o^ +/- 5°, 180° +/- 4° for the hip, and 140 ^o^ +/- 4°, 145 ^o^ +/- 4°, 160° +/- 4° for the tarsus, respectively. The median and mean standard error between baseline data for translation were 0.191 and 0.426 mm, respectively. The median and mean standard error between baseline data for rotation were 0.321° and 0.573°, respectively. The mean +/- standard error values of total translation, internal-external rotation, varus-valgus, and flexion-extension are summarized in Table 1. *P*-values for comparisons between groups are summarized in Table 2.

**Table 1:** Mean +/- standard error values for total translation, internal rotation, varus, and flexion.

	**Stifle Condition**	**125^**o**^**	**135^**o**^**	**145^**o**^**
Total Translation (mm)	CrCL-INTACT	1.459 ± 0.338	1.187 ± 0.165	0.970 ± 0.156
	CrCL-DEFICIENT	13.798 ± 1.651	13.488 ± 1.343	12.136 ± 1.259
	TAS	2.565 ± 0.303	2.341 ± 0.262	2.019 ± 0.233
Internal rotation (degrees)	CrCL-INTACT	2.769 ± 0.949	2.733 ± 0.757	1.816 ± 0.457
	CrCL-DEFICIENT	13.920 ± 2.073	12.966 ± 1.731	8.503 ± 1.816
	TAS	4.746 ± 0.805	4.586 ± 1.154	4.160 ± 1.116
Varus (degrees)	CrCL-INTACT	1.100 ± 0.331	0.744 ± 0.181	0.661 ± 0.245
	CrCL-DEFICIENT	5.517 ± 1.059	4.406 ± 0.907	4.094 ± 0.953
	TAS	3.351 ± 0.630	3.036 ± 0.497	1.998 ± 0.324
Flexion (degrees)	CrCL-INTACT	0.491 ± 0.185	0.338 ± 0.064	0.496 ± 0.191
	CrCL-DEFICIENT	3.054 ± 0.895	3.392 ± 0.808	3.380 ± 0.588
	TAS	1.180 ± 0.223	0.870 ± 0.238	0.881 ± 0.143

*Total translation is measured in mm, internal rotation, varus and flexion are measured in degrees. CrCL, cranial cruciate ligament; TAS, Titanium-alloy Anchor System*.

**Table 2:** Summarized *p*-values for differences in total translation, internal rotation, varus, and flexion between groups.

	**Stifle Condition**	**125^**o**^**	**135^**o**^**	**145^**o**^**
Total translation	CrCL-INTACT–CrCL-DEFICIENT	0.00007^*^	0.00002^*^	0.00002^*^
	CrCL-DEFICIENT–TAS	0.00005^*^	0.00001^*^	0.00002^*^
	CrCL-INTACT–TAS	0.02065^*^	0.00457^*^	0.00262^*^
Internal rotation	CrCL-INTACT– CrCL-DEFICIENT	0.002^*^	0.001^*^	0.004^*^
	CrCL-DEFICIENT–TAS	0.003^*^	0.003^*^	0.067
	CrCL-INTACT–TAS	0.146	0.083	0.036^*^
Varus	CrCL-INTACT– CrCL-DEFICIENT	0.005^*^	0.004^*^	0.011^*^
	CrCL-DEFICIENT–TAS	0.162	0.316	0.085
	CrCL-INTACT–TAS	0.011^*^	0.002^*^	0.005^*^
Flexion	CrCL-INTACT– CrCL-DEFICIENT	0.029^*^	0.005^*^	0.001^*^
	CrCL-DEFICIENT–TAS	0.087	0.022^*^	0.003^*^
	CrCL-INTACT–TAS	0.094	0.057	0.011^*^

*Statistical significance was set at <0.05. CrCL, cranial cruciate ligament*.

* *Marks a significant test result. TAS, Titanium-alloy Anchor System*.

**Figure 3:**
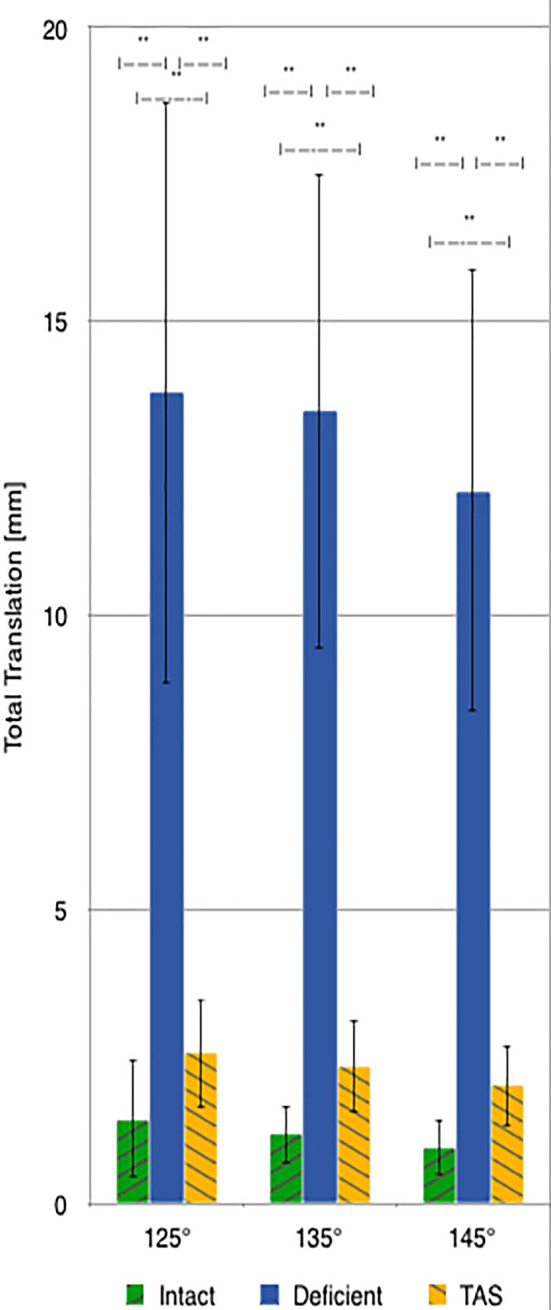
Mean values of total translation with the standard deviation bars applied. Comparisons between two groups that are significantly different from one another are indicated with a horizontal bar and double asterisks.

There was a significant increase in total translation between the CrCL-intact and CrCL-deficient groups at all three stifle angles. There was a significant decrease in total translation at all stifle angles when comparing the stifles with the TAS applied and CrCL-deficient groups. Total translation of the TAS group was greater than the CrCL-intact group at all stifle angles, which remained significant (Figure 3).

**Figure 4:**
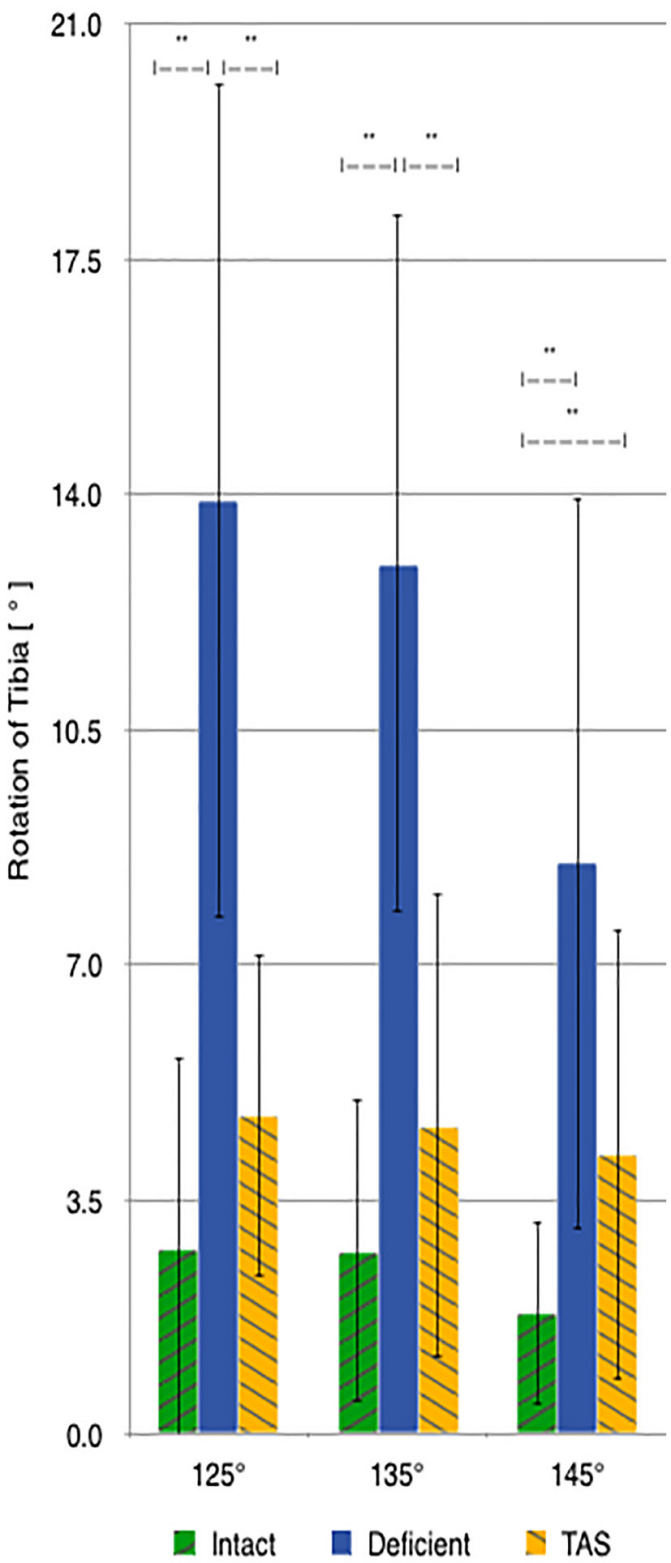
Mean values of internal-external rotation with the standard deviation bars applied. Comparisons between two groups that are significantly different from one another are indicated with a horizontal bar and double asterisks.

There was a significant increase in the amount of internal rotation present between the CrCL-intact and CrCL-deficient groups at all stifle angles. The TAS resulted in decreased internal rotation compared to the CrCL-deficient groups. This decrease was significant at stifle angles of 125° and 135°, but not at 145°. The difference in internal rotation between CrCL-intact and TAS groups was not different at 125° and 135°, but remained significantly increased in the TAS group at 145° (Figure 4). An additional comparison of internal rotation in the CrCL-deficient stifles was performed between each of the three stifle angles due to the overall smaller magnitude of internal rotation noted at 145° compared to either 125° or 135°; however, this difference was not significant. *P*-values for comparisons between 125° and 135°, 125° and 145°, and 135° and 145° were 0.392, 0.065, and 0.077, respectively.

A significant increase in varus motion was seen at all stifle angles when comparing the CrCL-intact and CrCL-deficient groups. Varus motion of the TAS group was significantly increased compared to the CrCL-intact group at all stifle angles.

## Discussion

The results of this study demonstrated that the TAS reduced the total translation and internal rotation of the tibia relative to the femur when applied to the CrCL-deficient stifle under the tested loading conditions. The TAS differed from the relative motion seen in the CrCL-intact stifle, thus rejecting the second part of our hypothesis.

Following transection of the CrCL, the range of mean increases for total translation of the tibia relative to the femur was 11.1–12.3 mm at the measured angles. This is similar to what is previously reported (8). Following the TAS, the total translation of the tibia relative to the femur was reduced, resulting in <1.2 mm of difference in translation between the CrCL-intact and TAS groups. Although this discrepancy between the CrCL-intact and TAS groups was significant at all angles measured, the clinical relevance of this translation is unknown. A previous report of clinical cases using this implant system reported satisfactory improvements in lameness by both veterinarians and owners. To the authors knowledge, there is no published data from which we can extrapolate to determine how this small amount of translation would affect live patients. Moreover, residual translation should be standardized against patient size as 1 mm of residual translation may have a differing clinical impact if it were to occur in a giant vs. a toy-breed dog.

Although tibial translation does not imply meniscal translation, it could be relevant to examine the effects of this residual translation on the incidence of postliminary meniscal tears. Since normal menisci can translate up to 13 mm, investigating whether a normal meniscus could withstand this residual tibial translation would be interesting; such conclusions are beyond the scope of this study (22). Furthermore, the data collected under this testing protocol represent static loading conditions, and are not representative of the complex movements of the canine stifle during the various activities of live dogs or cyclic loading over time.

Although transection of the CrCL caused a universal increase in internal tibial rotation at all three stifle angles, the smallest mean increase in internal rotation occurred at 145°, compared to 125° and 135°. Although the differences between 145° and each of the other angles was not significant, it did approach statistical significance with *p*-values nearing 0.05 compared to the *p*-value for comparing 125° and 135°, which was much larger. This difference may be explained by the lateral collateral ligament becoming more taut and resisting internal rotation of the tibia as the stifle is extended (23). However, tension within the lateral collateral ligament was not evaluated in this study, and therefore a definitive cause for the lesser internal rotation noted at 145° cannot be identified. Following the TAS, the amount of internal rotation decreased to within 2.4° of the CrCL-intact stifle group for each measured angle. The amount of internal rotation present was not significantly different between the intact and the TAS groups at angles of 125° and 135°, but was significantly greater at 145°. The lack of significance between groups for internal rotation can also be due to type II error.

Previous methods of extracapsular repair have demonstrated external rotation of the tibia relative to the femur resulting from over-tightening of the prosthesis (24). This external rotation of the tibia is theorized to contribute to the increased pressures within the lateral compartment of the stifle (24). Over-tightening of the prosthesis is performed consciously due to the expected loosening of the material used over time, as well as to mitigate cranial tibial translation. This is performed due to the perception that this is required for the surgery to be successful. The selected titanium link that connects the femoral and tibial bone anchors minimizes the amount of internal tibial rotation without resulting in external rotation beyond what would be expected in the CrCL-intact stifle. UHMWPE loops elongate less over time than other materials used in alternative methods of extracapsular repair, and this could be a possible benefit of the implant design that avoids increasing the pressures within the lateral compartment (25). Such a conclusion is beyond the scope of static loading conditions performed in this experiment, but another study has demonstrated results that support a similar inference (26).

Varus motion was increased at all stifle angles measured when comparing the CrCL-intact and CrCL-deficient groups, and was subsequently reduced with the TAS to within a mean of 2.3° of the CrCL-intact stifle. In contrast, previous studies on extracapsular repair methods reported increased pressures within the lateral compartment corresponding to increased valgus resulting from overtightening of the applied prosthesis (27). The increased pressure within the lateral compartment results in articular cartilage damage and lateral meniscal tears, which are reported with an increased frequency (28, 29). The TAS implant design theoretically avoids overtightening the prosthesis by selecting the link size that most closely corresponds to the remaining distance between both the tibial and femoral anchor UHMWPE loops. Choosing a link size less than the remaining distance would result in an overtight prosthesis, while choosing a link size greater than the remaining distance would result in a lax prosthesis. Selecting the appropriate link avoids increasing pressure within the lateral compartment that may increase the risk for lateral meniscal damage. However, pressures within the lateral compartment were not measured in this study; therefore, conclusions about the effects of this technique in a live dog are unknown.

The pursuit of physiologic isometry is paramount in methods of extracapsular repair to avoid both laxity within the prosthesis and limited stifle motion. In this study, the TAS was not able to duplicate normal stifle motion and this discrepancy is likely due to the difficulties in achieving physiologic isometry (30–33). It is unlikely that any form of extracapsular repair will replicate the functions of the CrCL because of the complexity of its physiologic isometry (31, 33).

In this investigation, a load was applied directly to the head of the femur instead of potting the femur in polymethyl methacrylate. We believe this method of eccentrically loading the pelvic limb allows unconstrained motion of the femur and tibia relative to one another, which better simulates normal weight-bearing of the canine limb (34). This is in contrast to previously reported testing methods (30, 35, 36).

The most significant limitations of this study are those related to study design and include the *ex-vivo* conditions, which limit the ability to extrapolate these results to the TAS *in vivo*. This is in part due to the absence of limb musculature. Also, materials used for simulating the patellar tendon and gastrocnemius were not the same (UHMWPE vs. metal cables, respectively) and could contribute unknown error to the results. The authors' reasoning for using different materials included consideration for the experimental setup: use of a metal cable with preformed loops would have required a larger hole to be drilled through the patella and could have resulted in patellar fracture, as occurred in one of the test specimens. Additionally, the metal cables are less pliable than the UHMWPE fiber, and therefore would have lifted the base of the patella cranially out of the trochlear groove. Metal cables were used to simulate the gastrocnemius due to ease of study design; each cable had a performed loop at either end that allowed equal distribution of tension between screw heads inserted in the femur. Furthermore, the stifles used in this study were considered healthy, thus the results may not be extrapolated directly to diseased stifles and associated periarticular tissues. An additional consideration is the small number of stifles used in this study.

The authors calculated changes in relative motion of the femur and tibia by manually establishing baseline data for each limb to be tested. This method has not been previously reported, and could serve to introduce error to the results if baseline data was not consistently established. In order to confirm the consistency of the baseline data, standard error measurements between baseline data points were calculated. It is important to note that any interpretation of the data in this study is limited by the standard error between baseline measurements; however, the mean standard error for baseline measurements was small (~0.5 mm). Additionally, measurements for this study were collected under static loading as opposed to cyclic loading, which more accurately simulates *in vivo* conditions.

In conclusion, the TAS resulted in reduced translation and internal rotation of the tibia relative to the femur compared to the CrCL-deficient stifle, but still differed from the CrCL-intact stifle. The differences between the TAS and CrCL-intact groups were small, and may result in clinically acceptable outcomes, but the effect of the residual translation and internal rotation is unknown. Additional studies evaluating the long-term clinical outcome of the TAS are required to investigate the effects of the TAS on the incidence of postliminary meniscal tears, maintenance of long-term stability, and progression of OA, compared to traditional extracapsular techniques.

## Data Availability Statement

The raw data supporting the conclusions of this article will be made available by the authors, without undue reservation.

## Author Contributions

CD and OL were responsible for the design of this study. CD, OL, and DSa were responsible for the statistical analysis. CD, OL, NM, DSa, KA, TP, and DSe were responsible for the preparation of the manuscript for submission. All authors contributed to the article and approved the submitted version.

## Conflict of Interest

OL is a paid consultant for KYON Veterinary Surgical Products. DS was an employee of KYON Veterinary Surgical Products. The remaining authors declare that the research was conducted in the absence of any commercial or financial relationships that could be construed as a potential conflict of interest.

## Footnotes

^1^Ruby Joint Stabilization System, KYON Veterinary Surgical Products, Zurich, Switzerland.

^2^KYON Veterinary Surgical Products, Zurich, Switzerland.

^3^Transducer Techniques, Temecula, CA, United States.

^4^FASTRAK: Polhemus, Colchester, VT, United States.

^5^Ethicon, Inc., a Johnson & Johnson company, Somerville, NJ, United States.

^6^FASTRAK: Polhemus, Colchester, VT, United States.

^7^MATLAB: The MathWorks Inc., Natick, MA, United States.
